# The association between hepatitis B virus and semen quality: a systematic review and meta-analysis

**DOI:** 10.1186/s12894-024-01424-9

**Published:** 2024-02-22

**Authors:** Yuting Xu, Kai Gan, Liqing Hou, Huawei Wang, June Cai, Liu Liu, Wen Wen, Meng Rao, Li Tang

**Affiliations:** 1 Department of Reproductive genetics, Yan ’an Hospital Affiliated to Kunming Medical University, Kunming, 650051 China; 2 Department of Organ Transplantation, The First Affiliated Hospital of Kunming Medical University, Kunming, 650032 China; 3 Department of Reproductive genetics, The First Affiliated Hospital of Kunming Medical University, 295 Xichang Road, Kunming, Yunnan Province 650032 China

**Keywords:** Hepatitis B virus, Semen quality, Meta-analysis, Systematic review

## Abstract

**Background:**

Some studies have suggested that hepatitis B virus (HBV) infection had a negative association with semen quality, but the conclusions have been inconsistent. The purpose of our study was to systematically assess the association between HBV infection and semen parameters.

**Methods:**

We searched electronic databases for studies published from January 1980 to August 2023. Eleven studies were included in the analysis. Primary outcomes were semen volume, sperm concentration, sperm morphology, sperm motility and sperm progressive motility. We also conducted a subgroup analysis between China and other countries.

**Result:**

Compared with the semen quality of HBV-negative men, HBV infection had a negative association with semen volume (MD: −0.20 mL, 95%CI: −0.32 to − 0.09, *P* = 0.0004), sperm concentration (MD: −4.46 × 10^6^/mL, 95%CI: −7.09 to − 1.84, *P* = 0.0009), sperm morphology (MD: −2.49%, 95%CI: −4.35 to − 0.64, *P* = 0.008), sperm motility (MD: −6.85%, 95%CI: −11.53 to − 2.18, *P* = 0.004), and sperm progressive motility (MD: −6.63%, 95%CI: −10.24 to − 3.02, *P* = 0.0003). However, HBV infection had no significant association with total sperm count (MD: −31.50 × 10^6^, 95%CI: −74.11 to 11.10, *P* = 0.15). The association between HBV and semen quality were inconsistent between the subgroups.

**Conclusion:**

HBV infection had a negative association with sperm concentration, motility, morphology, and semen volume. However, The association between HBV and total sperm count remain unclear. This metaanalysis suggests that we should pay attention to the adverse effect of HBV on sperm quality, and several studies have reported the relevant mechanisms. But due to the significant heterogeneity among studies on some semen parameters, further large and well-designed researches are needed before introducing clinical management recommendations.

**Supplementary Information:**

The online version contains supplementary material available at 10.1186/s12894-024-01424-9.

## Background

Infertility is a condition that the couple are unable to conceive after 12 months of regular unprotected intercourse [1]. And it has become the third-highest health hazard worldwide after cardiovascular disease and cancer [2]. Approximately 15% of married couples experience infertility, among which male infertility accounts for about 50%. Common factors leading to male infertility include reproductive system diseases and genetic and environmental factors [3]. Chronic viral infection is one cause of male infertility [4]. Hepatitis B virus (HBV) infection is a major global public health issue. According to WHO calculates, 295 million people worldwide had chronic hepatitis B in 2019, accounting for 3.8% of the global population [5]. HBV is highly contagious, mainly spread from mother to child or iatrogenic infection or sexual transmission. HBV mainly affects the liver but also exists in extrahepatic tissues, such as the testis, kidney and ovary [6]. HBV can cross the blood-testis barrier, invading spermatogonial cells, primary spermatocytes, sperm cells, and Sertoli cells [6]. A nationwide, population-based cohort study shows that HBV infection increases the incidence and risk of male infertility [7]. And semen quality is the cornerstone of evaluating male fertility [8].

However, the association between HBV and semen quality have been not clear. Studies have reported conflicting results on the association between HBV and semen quality. Some studies have shown that HBV infection significantly reduces sperm quality in men, including sperm concentration, sperm motility, and morphology [9, 10], while others did not detect significant differences [11]. We carried out this meta-analysis to clarify the association between HBV and semen quality. The results of this analysis will elucidate the association between HBV and male fertility and guide clinical research to solve the fertility needs of HBV-positive men.

## Methods

### Retrieval methods and inclusion criteria

This research was conducted according to the PROSPERO guideline (Registration ID CRD42022311270). We used the Meta-analysis of Observational Studies in Epidemiology consensus statement and PRISMA Statement as guidelines for the meta-analysis [12, 13].

We analyzed studies with at least one HBV-positive man as the case group and at least one HBV-negative man as the control group. To be included, a study must have covered at least one of the following outcomes: semen volume, sperm concentration, total sperm count, sperm morphology, sperm motility, and sperm progressive motility.

Exclusion criteria included the following: (1) The participants had received anti-HBV therapy. (2) The participants had liver cirrhosis caused by chronic HBV or hepatitis C virus (HCV) infection. (3) The participants were in the acute stage of HBV infection. (4) The participants also suffered from HCV, *Treponema pallidum* (TP), human immunodeficiency virus, or herpes simplex virus. (5) The participants had oligoasthenospermia or azoospermia with a clear cause (e.g., chromosome abnormalities, radiotherapy, chemotherapy, mumps, varicocele, or surgical history or congenital defects related to urology or the reproductive organs). (6) The study data could not be extracted. (7) The study was not published in the English language. (8) The publications were reviews, case reports, meetings, editorials, letters, or guidelines.

We included studies published from January 31, 1980, to August 1, 2023 and had no special requirements for the publication status or type of research. We combined Medical Subject Heading (MesH) Terms and Free-word Terms to retrieval those electronic databases: MEDLINE (by PubMed), The Cochrane Central Register of Controlled Trials (by CENTRAL), and EMBASE (by Ovid). Besides, we used the reference lists of publications included in this meta-analysis to manually retrieve additional studies that complied with the inclusion criteria. The following keywords were used: Hepatitis B, Hepatitis B Virus Infection, Hepatitis B virus, B virus, Hepatitis, Hepatitis B viruses, Viruses, Hepatitis B, Hepatitis Virus, Homologous Serum, Hepatitis B Surface Antigens, Hepatitis B Surface Antigen, HBsAg, Hepatitis B, Chronic, Chronic Hepatitis B Virus Infection, Chronic Hepatitis B, Hepatitis B Virus Infection, Chronic, Semen Analysis, Semen Analyses, Semen Quality Analysis, Analyses, Semen Quality, Analysis, Semen Quality, Quality Analyses, Semen, Semen Quality Analyses, Semen Quality, Qualities, Semen, Quality, Semen, Semen Qualities. We did not apply any other retrieve restrictions.

YTX and KG authors independently performed retrieval, screening, and data extraction tasks independently. Any disagreements were solved by consultation or the decision of another author (HWW or HM). Studies that complied with the inclusion criteria were separately read and screened in full.

### Data analysis

The extracted data included characteristics of study (first author, publication date, country, type of study) and characteristics of participant (recruitment period, number, age, use of assisted reproductive technology [ART]). The outcomes extracted were semen quality parameters (including semen volume, sperm concentration, total sperm count, sperm morphology, sperm motility, and sperm progressive motility). We used RevMan software (version 5.3) to create a risk of bias (quality) assessment chart. Because the articles we included were observational (case-control and cohort studies) rather than randomized controlled trials, we used the Newcastle–Ottawa Scale (NOS) to assess the risk of bias [14, 15]. The NOS evaluates study quality in the following three categories: (1) study population selection (0 to 4 points); (2) comparability between groups (0 to 2 points); (3) outcome measurements (0 to 3 points). The NOS scores range from 0 to 9 (low quality, ≤ 5 points; medium quality, 6 to 7 points; high quality, 8 to 9 points). Two authors (YTX and KG) independently scored studies using the NOS, and any controversy were solved by consensus or another author (HWW). We did not exclude studies from our analysis due to low quality evaluation scores.

We used RevMan software (version 5.5) and Stata software (version 16.0) for this meta-analysis. Because the outcome observation index of our study was continuous variable data, the units were the same among all included studies, and the mean difference (MD) and its 95% confidence interval (CI) were used to express the effect size. We used the heterogeneity index *I*^*2*^ and Q-test to test heterogeneity.

The P-value > 0.1 in Q-test indicated that heterogeneity was not statistically significant, and the results of multiple studies were considered statistically homogeneous. The P-value ≤ 0.1 in Q-test indicated that heterogeneity was statistically significant, and the results of multiple studies were considered statistically heterogeneous. The heterogeneity index *I*^2^ can quantitatively describe the degree of heterogeneity (*I*^2^ values of < 25%, 25–50%, 50–75%, and > 75%, represent mild, moderate, substantial, and considerable heterogeneity, respectively) [16]. When the *I*^2^ value > 50%, the included studies were considered significant heterogeneity, and a random-effects model was used. When the *I*^2^ value ≤ 50%, the included studies were considered homogeneous, and a fixed-effects model was used. We used the *Z*-test to test the overall effect. When the *P*-value of the *Z*-test ≤ 0.05, the pooled effect size was statistically significant. When the *P*-value of the *Z*-test > 0.05, the pooled effect size was not statistically significant. We conducted a subgroup analysis comparing participants in China and other countries using Stata software (Version 16.0). Additionally, we did a sensitivity analysis, eliminating one study at a time to investigate the sources of heterogeneity. Egger’s test was used to detect publication bias of included studies, a *P*-value < 0.1 indicated significant publication bias.

## Results

### Literature search

**Fig. 1:**
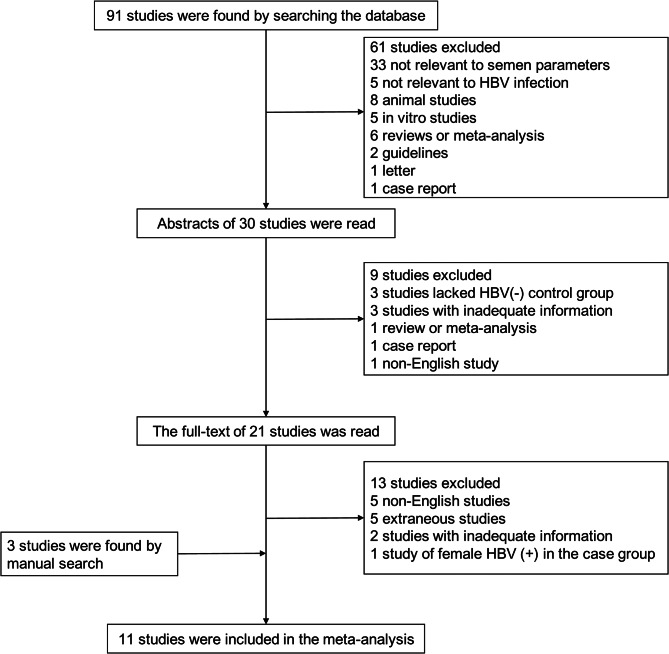
Flow chart

We retrieved 91 articles during an initial database search. By reading titles and abstracts, we excluded 70 studies that were unrelated, reviews, letters, or non-English language. The full texts of the remaining 21 articles were assessed, and eight met our study criteria. Another three articles were found by manual retrieval. Eleven articles met all criteria and were included in this meta-analysis. The detailed search steps are summarized in Fig. 1.

### Study characteristics and quality

This research included 1,291 HBV-positive male participants and 3,515 HBV-negative controls from 11 studies published from 2003 to 2020. Three were retrospective cohort studies [17–19], and the remaining eight were case-control studies [10, 20–26]. Among the included studies, six were conducted in China [17, 22–24, 26], two were in Italy [10, 18], and the remaining three were from Iran, Ghana, and France, respectively [19–21]. Nine studies included participants who received ART [17, 18, 21, 23–26], but the ART statuses of participants in two studies were unknown [20, 22]. Each included study was assessed for risk of bias using the NOS (Supplementary Fig. 1). According to the NOS scores, four studies were high quality, six were medium quality, and one was low quality. The study characteristics and quality evaluations are summarized in Table 1.

**Table 1:** Characteristics of included studies

Study	Year published	Country	Study design	Use of ART	Recruitment Period	HBV(+) group		Control group		Outcome	Guidelines for semen analysis	Newcaste-Ottawa score
						Patients	Age (years)	Patients	Age (years)			
Bu et al. [17]	2014	China	cohort study	Yes	2010–2012	20	36.7 ± 7.2	257	36 ± 7.4	semen volume, sperm concentration, motility, progressive motility rate, morphology	Unclear	Medium (6 points)
F. Lorusso et al. [10]	2009	Italy	Case control	Yes	2004–2008	30	36.9 ± 6.1	130	36 ± 4.1	semen volume, sperm concentration, progressive motility rate, morphology	WHO 1999	Low (5 points)
G. Cito et al. [18]	2019	Italy	cohort study	Yes	2011–2018	66	36.7 ± 6.5	68	38.7 ± 4.4	semen volume, total sperm count, sperm concentration, progressive motility rate, morphology	WHO 2010	High (8 points)
Karamolahi et al. [20]	2019	Iran	Case control	Unclear	2003–2014	112	36.7 ± 7.5	112	34.4 ± 7.8	semen volume, total sperm count, sperm motility, morphology,	WHO 1999	High (8 points)
P. Oger et al. [21]	2011	France	Case control	Yes	2005–2008	32	34.7 ± 5.0	64	35.3 ± 6.3	sperm concentration, progressive motility rate, morphology, motility	WHO 1992	Medium (7 points)
Qian et al. [22]	2016	China	Case control	Unclear	2015–2015	30	…	30	…	semen volume, sperm concentration, progressive motility rate, motility, morphology	Unclear	Medium (6 points)
Shi et al. [23]	2014	China	Case control	Yes	2008–2012	136	32.9 ± 4.7	272	32.6 ± 5.0	sperm concentration, progressive motility rate, morphology	WHO 2010	Medium (7 points)
VCY Lee et al. [24]	2010	China	Case control	Yes	2004–2008	154	…	1473	…	sperm concentration, progressive motility rate, morphology	Unclear	Medium (6 points)
Wang et al. [25]	2021	China	Case control	Yes	2016–2020	227	34.8 ± 4.9	454	34.7 ± 4.7	semen volume, sperm concentration, progressive motility rate, morphology	WHO 1999	High (8 points)
Yakass, M.B et al. [19]	2016	Ghana	cohort study	Yes	2013–2015	27	…	196	…	sperm concentration, progressive motility rate, morphology	WHO 2010	Medium (6 points)
Zhou et al. [26]	2011	China	Case control	Yes	2008–2009	457	33.4 ± 4.8	459	33.5 ± 4.8	semen volume, total sperm count, sperm concentration, progressive motility rate, morphology	WHO 1999	High (8 points)

### Meta-analysis results

#### Association between HBV infection with semen volume

**Fig. 2:**
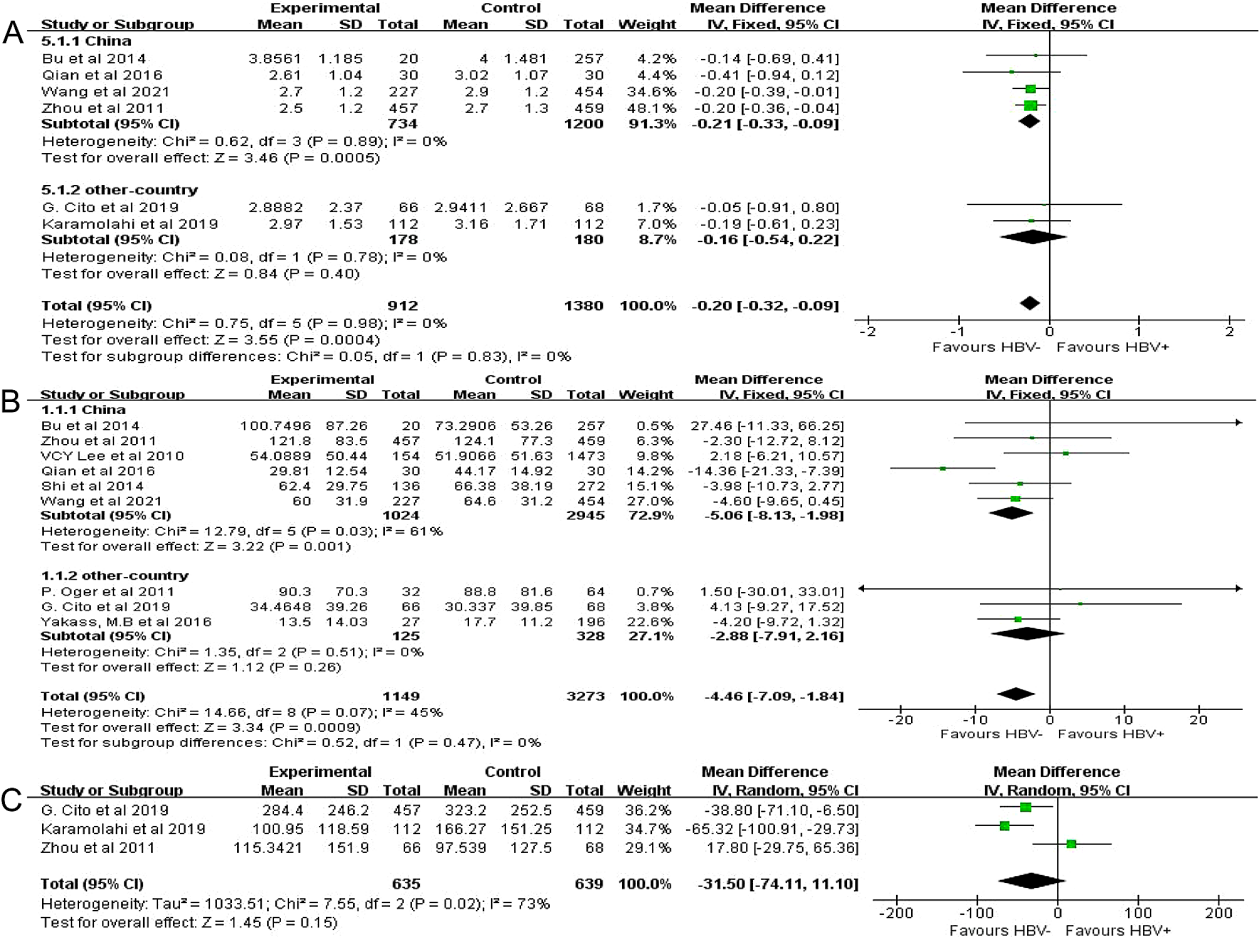
Effects of hepatitis B virus (HBV) on semen volume **(A)**, sperm concentration **(B)**, and total sperm count **(C)**

Six studies involving 2,292 participants analyzed the association between HBV infection with semen volume. HBV infection had a negative association with semen volume (MD: −0.20 mL, 95%CI: −0.32 to − 0.09, *P* = 0.0004; Fig. 2A). The heterogeneity test did not detect heterogeneity between studies (*P* = 0.98, *I*^2^ = 0%), and the sensitivity analysis showed low sensitivity and relatively stable results (Supplemental Fig. 2A). No publication bias was detected (*P* = 0.930; Supplemental Fig. 3A).

Next, the studies were divided based on their locations into two subgroups, China and other countries. In the China subgroup, there were four studies with no significant heterogeneity between them (*P* = 0.89, *I*^2^ = 0%). HBV infection had a negative association with semen volume (MD: −0.21 mL, 95%CI: −0.33 to − 0.09, *P* = 0.0005; Fig. 2A). In the other-country subgroup, there were two studies with no significant heterogeneity between them (*P* = 0.78, *I*^2^ = 0%). HBV infection had no significant association with semen volume in this subgroup (MD: −0.16 mL, 95%CI: −0.54 to 0.22, *P* = 0.40; Fig. 2A).

#### Association between HBV infection with sperm concentration

Nine studies involving 4,422 participants analyzed the association between HBV infection with sperm concentration. HBV infection had a negative association with sperm concentration (MD: −4.46 × 10^6^/mL, 95%CI: −7.09 to − 1.84, *P* = 0.0009; Fig. 2B). The heterogeneity test showed that the included studies were moderately heterogeneous (*P* = 0.07, *I*^2^ = 45%). The sensitivity analysis showed low sensitivity and relatively stable results (Supplemental Fig. 2B), and no publication bias was detected (*P* = 0.173) (Supplemental Fig. 3B).

In the China subgroup, there were six studies with substantial heterogeneity between them (*P* = 0.03, *I*^2^ = 61%). HBV infection had a negative association with sperm concentration (MD: −5.06 × 10^6^/mL, 95%CI: −8.13 to − 1.98, *P* = 0.001; Fig. 2B). In the other-country subgroup, there were three studies with no heterogeneity between them (*P* = 0.51, *I*^2^ = 0%). HBV infection had no significant association with sperm concentration (MD: −2.88 × 10^6^/mL, 95%CI: −7.91 to 2.16, *P* = 0.26; Fig. 2B).

#### Association between HBV infection with total sperm count

Three studies involving 1,274 participants analyzed the association between HBV infection with total sperm count. HBV infection had no significant association with total sperm count (MD: −31.50 × 10^6^, 95%CI: −74.11 to 11.10, *P* = 0.15; Fig. 2C). There was substantial heterogeneity in the included studies (*P* = 0.02, *I*^2^ = 73%). The sensitivity was low, and the results were relatively stable (Supplemental Fig. 2C). No publication bias was detected (*P* = 0.423; Supplemental Fig. 3C).

Only one study assessed the effect of HBV infection on sperm count in the China subgroup; thus, the subgroup analysis was not performed.

#### Association between HBV infection with sperm morphology

**Fig. 3:**
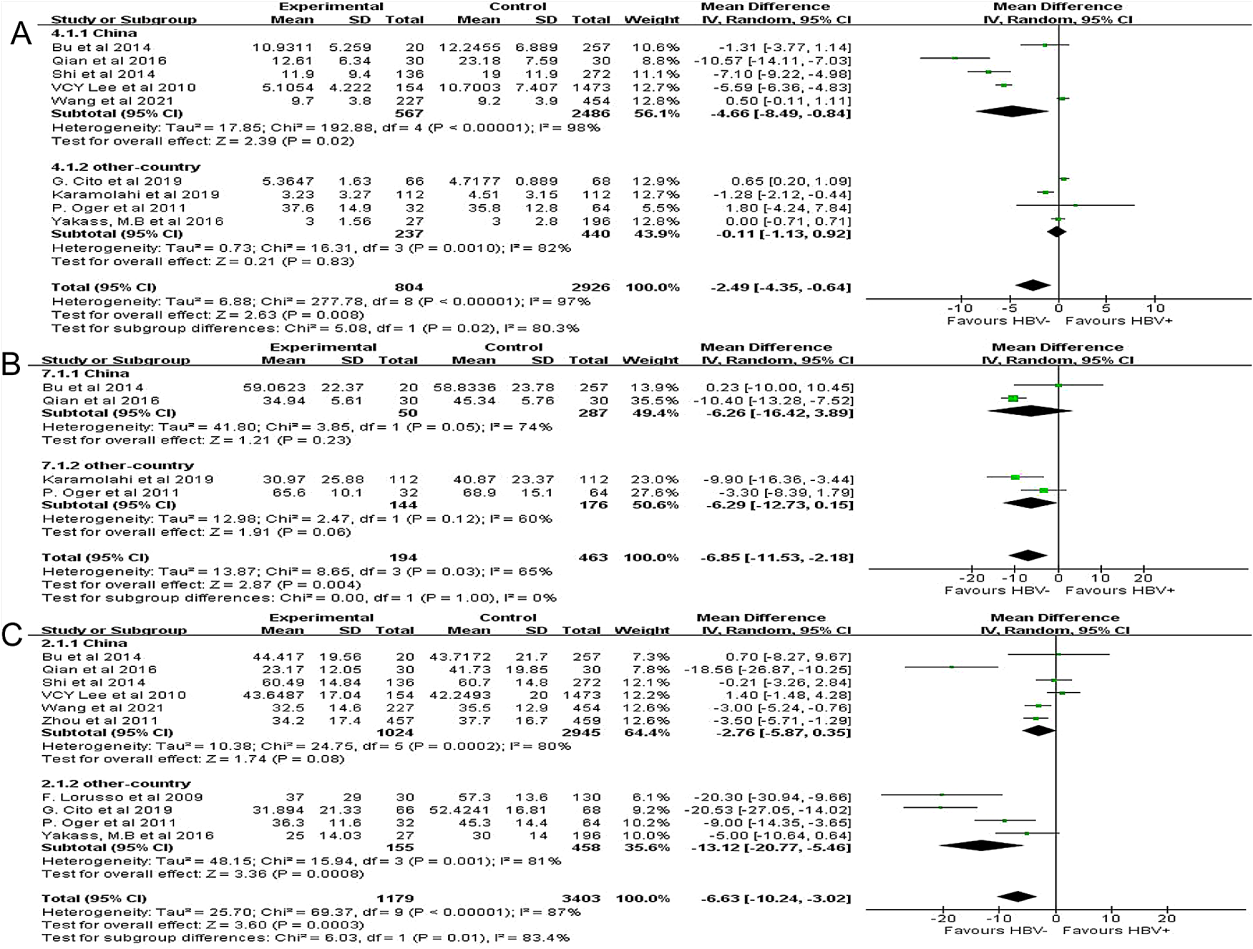
Effects of hepatitis B virus (HBV) on sperm morphology **(A)**, sperm motility **(B)** and sperm progressive motility **(C)**

Nine studies involving 3,730 participants analyzed the association between HBV infection with sperm morphology. HBV infection had a negative association with sperm morphology (MD: −2.49%, 95%CI: −4.35 to − 0.64, *P* = 0.008; Fig. 3A). The included studies were considerably heterogeneous (*P* < 0.00001, *I*^2^ = 97%). The sensitivity analysis showed low sensitivity and relatively stable results (Supplemental Fig. 2D), and no publication bias was detected (*P* = 0.195; Supplemental Fig. 3D).

In the China subgroup, there were five studies with considerable heterogeneity between them (*P* < 0.00001, *I*^2^ = 98%). HBV infection had a negative association with sperm morphology (MD: −4.66%, 95%CI: −8.49 to − 0.84, *P* = 0.02; Fig. 3A). In the other-country subgroup, there were four studies with considerable heterogeneity between them (*P* = 0.001, *I*^2^ = 82%). HBV infection had no significant association with sperm morphology (MD: −0.11%, 95%CI: −1.13 to 0.92, *P* = 0.83; Fig. 3A).

#### Association between HBV infection with sperm motility

Four studies involving 657 participants analyzed the association between HBV infection with sperm motility. HBV infection had a negative association with sperm motility (MD: −6.85%, 95%CI: −11.53 to − 2.18, *P* = 0.004; Fig. 3B). The included studies had substantial heterogeneity (*P* = 0.03, *I*^2^ = 65%). The sensitivity analysis showed low sensitivity, and the result was relatively stable (Supplemental Fig. 2E). No publication bias was detected (*P* = 0.280; Supplemental Fig. 3E).

In the China subgroup, there were two studies with substantial heterogeneity between them (*P* = 0.05, *I*^2^ = 74%). HBV infection had no significant association with sperm motility (MD: −6.26%, 95%CI: −16.42 to 3.89, *P* = 0.23; Fig. 3B). In the other-country subgroup, there were two studies with substantial heterogeneity between them (*P* = 0.12, *I*^2^ = 60%). HBV infection had no significant association with sperm motility (MD: −6.29%, 95%CI: −12.73 to 0.15, *P* = 0.06; Fig. 3B).

#### Association between HBV infection with sperm progressive motility

Ten studies involving 4,582 participants analyzed the association between HBV infection with sperm progressive motility. HBV infection had a negative association with sperm progressive motility (MD: −6.63%, 95%CI: −10.24 to − 3.02, *P* = 0.0003; Fig. 3C). The included studies were considerably heterogeneous (*P* < 0.00001, *I*^2^ = 87%). The sensitivity analysis showed low sensitivity, and the result was relatively stable (Supplemental Fig. 2F). Publication bias was detected (*P* = 0.046; Supplemental Fig. 3F).

In the China subgroup, there were six studies with considerable heterogeneity between them (*P* = 0.002, *I*^2^ = 80%). HBV infection had no significant association with sperm progressive motility (MD: −2.76%, 95%CI: −5.87 to 0.35, *P* = 0.08; Fig. 3C). In the other-country subgroup, there were four studies with considerable heterogeneity between them (*P* = 0.001, *I*^2^ = 81%). HBV infection had a negative association with sperm progressive motility in this subgroup (MD: −13.12%, 95%CI: −20.77 to − 5.46, *P* = 0.0008; Fig. 3C).

## Discussions

HBV is one of the most widely transmitted viruses in the world, especially in China, where the positive rate of hepatitis B surface antigen is 6.89%, and about 10% of the population of childbearing age are affected by HBV [27]. In general, male fertility depends on sperm quality, and there is no consensus on.

the association between HBV infection with semen quality parameters. As far as we know, this is the first try to make a systematic review and meta-analysis of the association between HBV infection with semen quality. Our meta-analysis included 11 studies with 1,291 HBV-positive men and 3,515 HBV-negative controls. The results showed that HBV infection had negative association with sperm concentration, motility, morphology, and semen volume but no significant association with total sperm count.

The mechanism of HBV infection affecting sperm quality mainly involves the changes of host genome and immune inflammatory response. As early as 1985, Hadchouel et al. [28] discovered that HBV DNA could be integrated into sperm chromosomes, and HBV infection could be transmitted vertically through the germ cell. In 2003, Huang et al. [29] discovered an obvious increase in the proportion of sperm chromosome abnormalities in HBV-positive patients, which was attributed to the insertion of HBV DNA or damaged genetic material into sperm chromosomes by HBV. HBV DNA can integrate into multiple sites on sperm chromosomes without specificity. If HBV DNA is integrated into reproductive stem cells, repeated copying and DNA rearrangement can occur, which increases the instability of sperm chromosomes and leads to abnormalities, such as chromosome aneuploidy, fracture, deletion, and smashing [29], consequently reducing sperm quality and the normal sperm morphology ratio. Moretti et al. [11] also confirmed that the apoptosis and necrosis of spermatozoa in HBV infected patients exceeded the normal range, which was consistent with our study.

The male reproductive system has a natural immune response, and sperm exposed to HBV may produce high levels of reactive oxygen species (ROS), resulting in lipid peroxidation [30]. Excessive ROS production can cause serious oxidative damage to the sperm cell membrane and DNA, resulting in the loss of sperm DNA fragments and cell membrane integrity [31]. Lipid peroxidation of the sperm cell membrane can also lead to increased membrane fluidity and permeability, affecting intracellular and extracellular ion concentration and ion flow [30], thus affecting sperm motility, sperm activation, acrosome reaction, and Ovum fertilization [32]. Sperm exposed to HBV can activate mitochondrial apoptosis by activating the Bax/Bcl2 signaling pathway, which may then activate apoptosis mediated by the AIF/Endo G signaling pathway [33], resulting in decreased sperm counts and fertilization ability. Our meta-analysis indicated that male HBV infection had no significant association with sperm count. However, only three studies assessed this outcome. Among them, Karamolahi et al. [20] and Zhou et al. [26] found that HBV infection significantly reduced sperm count, and Cito et al. [18] did not detect an obvious effect on sperm count. The small number of included studies may be the reason for the lack of significance in our meta-analysis, so this result should be carefully interpreted.

A significant publication bias was detected in 10 studies on the effect of HBV on progressive sperm motility. It is possible that some authors who do not detect significant results do not attempt to publish the paper or publish in local non-English journals, which could be the cause of publication bias [34].

In addition, we detected significant statistical heterogeneity among these studies during the heterogeneity test, so we conducted subgroup analyses, grouping the studies by country. The heterogeneity of the China subgroup was generally higher than that of the overall heterogeneity, and the heterogeneity of the other-country subgroup was generally lower than that of the overall heterogeneity. Therefore, different countries may be one of the factors leading to heterogeneity. In the China subgroup, HBV infection had a negative association with semen volume, sperm concentration and sperm morphology but no significant association with sperm motility and sperm progressive motility. In the other-country subgroup, HBV infection had a negative association with sperm progressive motility but no significant association with semen volume, sperm concentration, sperm morphology, and sperm motility. Therefore, HBV infection had inconsistent association with semen quality between the two subgroups. The reasons for this inconsistency are likely to be complicated, but the most likely reason is that the sample size in each subgroup became smaller after grouping, which reduces the statistical power of the analysis and requires careful interpretation. Additionally, racial differences in semen quality have been reported among the general population. White men have higher semen volume but lower sperm count and semen concentration than Asian men [35].

The association between HBV infection with semen quality may vary depending on the baseline semen quality. Finally, there are significant regional differences in HBV genotype distribution as well as significant differences among dominant HBV genotypes in different countries and regions [36]. HBV genotype is closely related to the progression, clinical manifestation, and prognosis of hepatitis B [37]. The effects of HBV genotype on affinity and pathogenicity to sperm warrant further study.

There were some limitations to consider. First of all, the number of studies included was not large enough. Although only 11 studies were included, semen parameters were reflected in many aspects, including count, motility, and morphology, and not all studies reported all semen parameters. Second, there was no control for confounding factors. In particular, no subgroup classification of infertile and fertile individuals. Other factors that may influence semen parameters, including race, age, body mass index, medical history, dietary habits, and lifestyle, were not controlled for in some studies. Third, all included studies were retrospective cohort or case–control studies, making it impossible to prove a causal relationship. Finally, some articles were of poor quality and lacked detailed inclusion and exclusion criteria.

## Conclusion

Our findings suggest that HBV infection had negative association with sperm concentration, motility, morphology, and semen volume, but the association with sperm count remains unclear. In addition, the association between HBV infection with semen quality was inconsistent between the China subgroup and the other-country subgroup. Significant between-study heterogeneity was detected for some semen parameters, and more research is needed to clarify the effect of HBV on semen quality. Further, clarifying the relationship between HBV infection and male infertility is necessary to provide theoretical guidance for the treatment of male HBV infection with infertility.

### Electronic supplementary material

Below is the link to the electronic supplementary material.

Supplementary Material 1

Supplementary Material 2

Supplementary Material 3

Supplementary Material 4

Supplementary Material 5

Supplementary Material 6

## Data Availability

Data openly available in a public repository.
