# Supplementary figures and images for: The association between hepatitis B virus and semen quality: a systematic review and meta-analysis
Source: BMC Urol. 2024 Feb 22;24:47. doi: 10.1186/s12894-024-01424-9 (PMC10885473; doi:10.1186/s12894-024-01424-9)

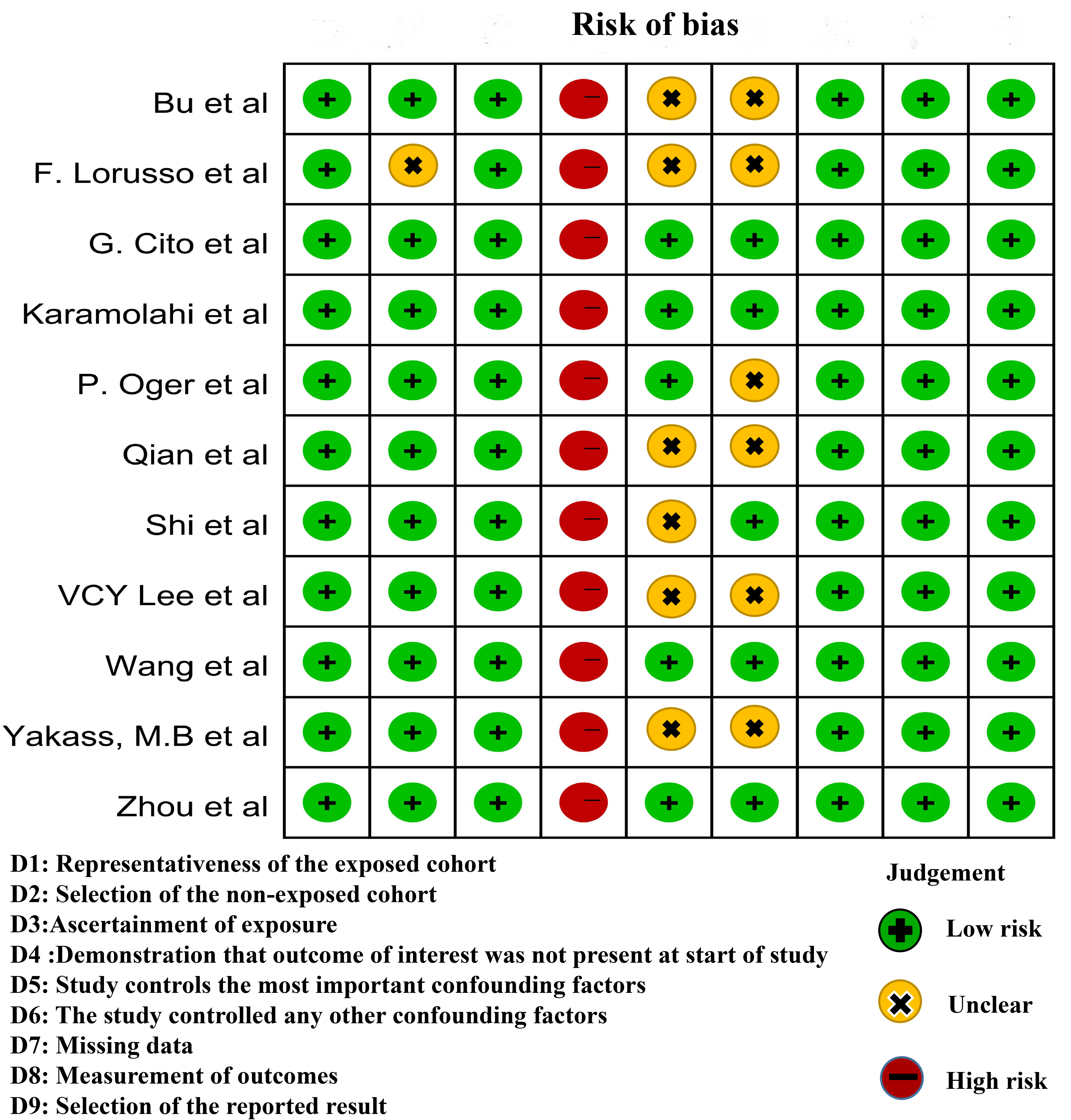

Supplement: Supplementary file 1 — Supplementary Material 1 [file 12894_2024_1424_MOESM1_ESM.jpg]

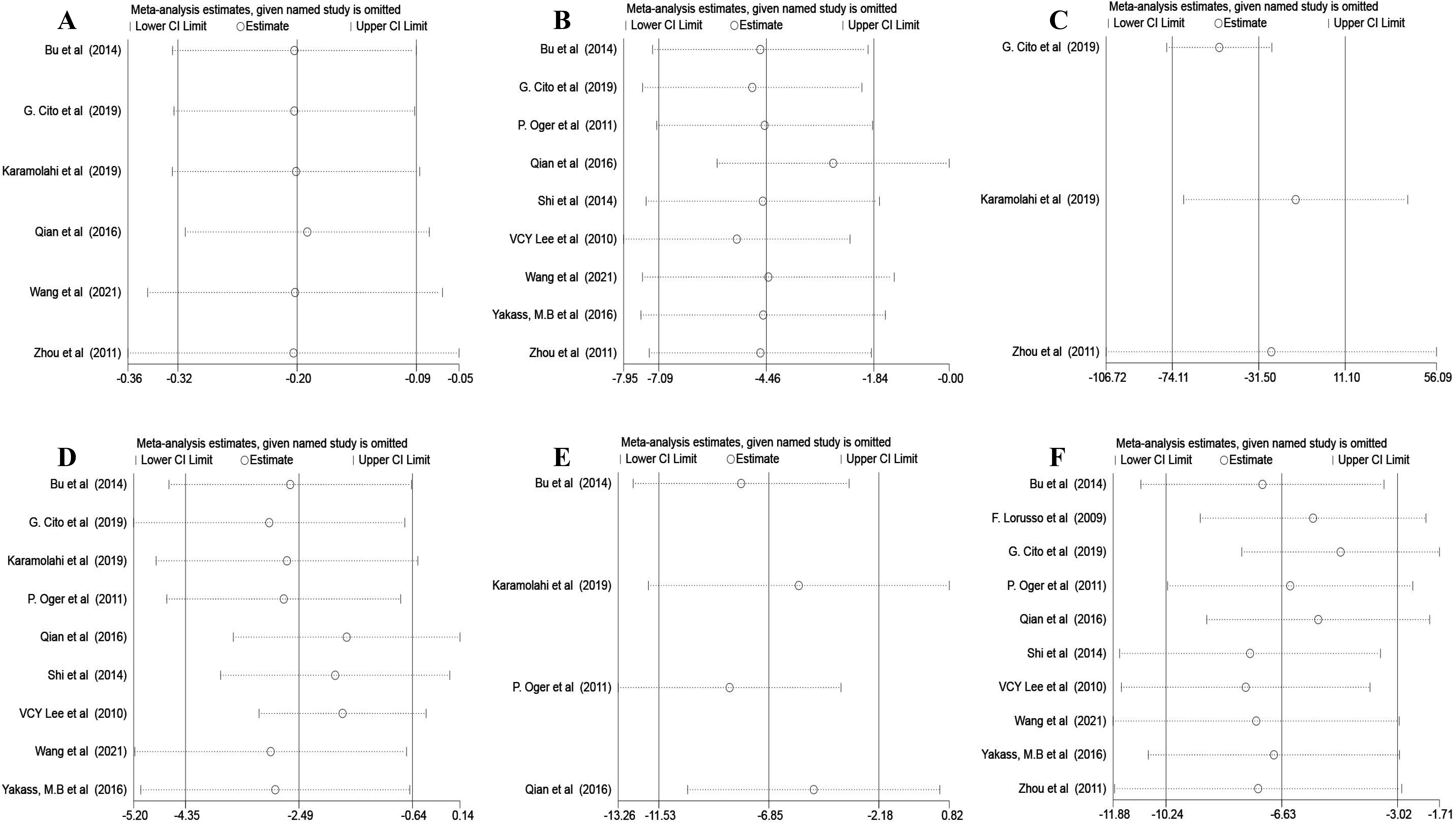

Supplement: Supplementary file 2 — Supplementary Material 2 [file 12894_2024_1424_MOESM2_ESM.jpg]

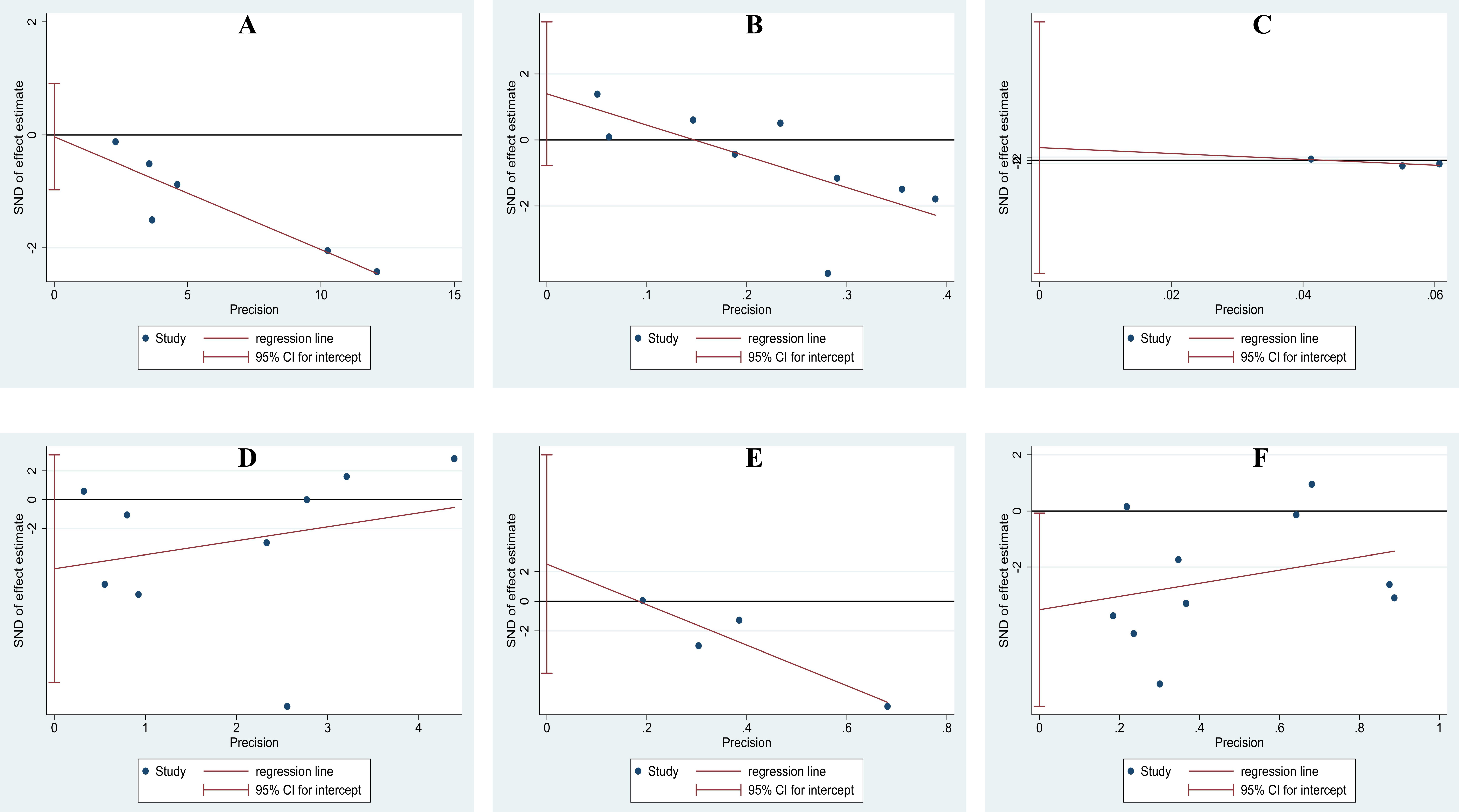

Supplement: Supplementary file 3 — Supplementary Material 3 [file 12894_2024_1424_MOESM3_ESM.jpg]
