# Supplementary material for: The association between hepatitis B virus and semen quality: a systematic review and meta-analysis
Source: BMC Urol. 2024 Feb 22;24:47. doi: 10.1186/s12894-024-01424-9 (PMC10885473; doi:10.1186/s12894-024-01424-9)
Supplement: Supplementary file 5 — Supplementary Material 5 [file 12894_2024_1424_MOESM5_ESM.docx]

Supplemental Figure 1: Risk of bias summary review authors' judgements about each risk of bias item for each included study

Supplemental Figure 2: Sensitivity analysis of the effect of hepatitis B virus (HBV) on semen quality (A: semen volume, B: sperm concentration, C: total sperm count, D: sperm morphology, E: sperm motility, F: sperm progressive motility)

Supplemental Figure 3: Egger test of the effect of hepatitis B virus (HBV) on semen quality (A: semen volume, B: sperm concentration, C: total sperm count, D: sperm morphology, E: sperm motility, F:sperm progressive motility)

Supplementary Table S1 : PICOS table

Supplementary Table S2 : Prisma Checklist
