# Supplementary material for: The association between hepatitis B virus and semen quality: a systematic review and meta-analysis
Source: BMC Urol. 2024 Feb 22;24:47. doi: 10.1186/s12894-024-01424-9 (PMC10885473; doi:10.1186/s12894-024-01424-9)
Supplement: Supplementary file 6 — Supplementary Material 6 [file 12894_2024_1424_MOESM6_ESM.docx]

| **Section and Topic** | **Item #** | **Checklist item** | **Location where item is reported** |
| --- | --- | --- | --- |
| **TITLE** | | |  |
| Title | 1 | The association between hepatitis B virus and semen quality:a systematic review and meta-analysis |  |
| **ABSTRACT** | | |  |
| Abstract | 2 | Background: Some studies have suggested that hepatitis B virus (HBV) infection had a negative association with semen quality, but the conclusions have been inconsistent. The purpose of our study was to systematically assess the association between hepatitis B virus and semen parameters.  Methods: We searched electronic databases for studies published from January 1980 to August 2023. Eleven studies were included in the analysis. Primary outcomes were semen volume, sperm concentration, sperm morphology, sperm motility and sperm progressive motility. We also conducted a subgroup analysis between China and other countries.  Result: Compared with the semen quality of HBV-negative men, HBV infection had a negative association with semen volume (MD: −0.20 mL, 95%CI: −0.32 to −0.09, P = 0.0004), sperm concentration (MD: −4.46×106/mL, 95%CI: −7.09 to −1.84, P = 0.0009), sperm morphology (MD: −2.49%, 95%CI: −4.35 to −0.64, P = 0.008), sperm motility (MD: −6.85%, 95%CI: −11.53 to −2.18, P = 0.004), and sperm progressive motility (MD: −6.63%, 95%CI: −10.24 to −3.02, P = 0.0003). However, HBV infection had no significant association with total sperm count (MD: −31.50×106, 95%CI: −74.11 to 11.10, P = 0.15). The association between HBV and semen quality were inconsistent between the subgroups.  Conclusion: HBV infection had a negative association with sperm concentration, motility, morphology, and semen volume. However, The association between HBV and total sperm count remain unclear. This meta‑analysis suggests that we should pay attention to the adverse effect of HBV on sperm quality, and several studies have reported the relevant mechanisms. But due to the significant heterogeneity among studies on some semen parameters, further large and well-designed researches are needed before introducing clinical management recommendations. |  |
| **INTRODUCTION** | | |  |
| Rationale | 3 | Some studies have suggested that hepatitis B virus (HBV) infection had a negative association with semen quality, but the conclusions have been inconsistent. |  |
| Objectives | 4 | To systematically assess the association between hepatitis B virus and semen parameters. |  |
| **METHODS** | | |  |
| Eligibility criteria | 5 | Inclusion Criteria:We analyzed studies with at least one HBV-positive man as the case group and at least one HBV-negative man as the control group. To be included, a study must have covered at least one of the following outcomes: semen volume, sperm concentration, total sperm count, sperm morphology, sperm motility, and sperm progressive motility.  Exclusion criteria included the following: (1) The participants had received anti-HBV therapy. (2) The participants had liver cirrhosis caused by chronic HBV or hepatitis C virus (HCV) infection. (3) The participants were in the acute stage of HBV infection. (4) The participants also suffered from HCV, Treponema pallidum (TP), human immunodeficiency virus, or herpes simplex virus. (5) The participants had oligoasthenospermia or azoospermia with a clear cause (e.g., chromosome abnormalities, radiotherapy, chemotherapy, mumps, varicocele, or surgical history or congenital defects related to urology or the reproductive organs). (6) The study data could not be extracted. (7) The study was not published in the English language. (8) The publications were reviews, case reports, meetings, editorials, letters, or guidelines.  Hepatitis B group were divided according to whether or not they were positive for HBsAg. |  |
| Information sources | 6 | We [combine](javascript:;)d Medical Subject Heading (MesH) Terms and Free-word Terms to retrieval those electronic databases: MEDLINE (by PubMed), The Cochrane Central Register of Controlled Trials (by CENTRAL), and EMBASE (by Ovid).  Reference:  Bu Z, Kong H, Li J et al: Effect of male hepatitis B virus infection on outcomes of in vitro fertilization and embryo transfer treatment: insights from couples undergoing oocyte donation. International journal of clinical and experimental medicine 2014, 7(7):1860-6.  Lorusso F, Palmisano M, Chironna M et al: Impact of chronic viral diseases on semen parameters. Andrologia 2010, 42(2):121-6.  Cito G, Coccia ME, Fucci R et al: Hepatitis B Surface Antigen Seropositive Men in Serodiscordant Couples: Effects on the Assisted Reproductive Outcomes. The world journal of men's health 2021, 39(1):99-106.  Karamolahi S, Yazdi RS, Zangeneh M et al: Impact of hepatitis B virus and hepatitis C virus infection on sperm parameters of infertile men. International journal of reproductive biomedicine 2019, 17(8):551-56.  Oger P, Yazbeck C, Gervais A et al: Adverse effects of hepatitis B virus on sperm motility and fertilization ability during IVF. Reproductive biomedicine online 2011, 23(2):207-12.  Qian L, Li Q, Li H: Effect of hepatitis B virus infection on sperm quality and oxidative stress state of the semen of infertile males. American journal of reproductive immunology (New York, NY : 1989) 2016, 76(3):183-5.  Shi L, Liu S, Zhao W et al: Hepatitis B virus infection reduces fertilization ability during in vitro fertilization and embryo transfer. Journal of medical virology 2014, 86(7):1099-104.  Lee VC, Ng EH, Yeung WS et al: Impact of positive hepatitis B surface antigen on the outcome of IVF treatment. Reproductive biomedicine online 2010, 21(5):712-7.  Wang Z, Liu W, Zhang M et al: Effect of Hepatitis B Virus Infection on Sperm Quality and Outcomes of Assisted Reproductive Techniques in Infertile Males. Frontiers in medicine 2021, 8:744350.  Yakass MB, Woodward BJ, Otoo MA et al: Prevalence of blood borne viruses in IVF: an audit of a fertility Centre. JBRA assisted reproduction 2016, 20(3):132-6.  Zhou XP, Hu XL, Zhu YM et al: Comparison of semen quality and outcome of assisted reproductive techniques in Chinese men with and without hepatitis B. Asian journal of andrology 2011, 13(3):465-9.  Last searched data: August 1, 2023 |  |
| Search strategy | 7 | (((Hepatitis B) OR (Hepatitis B Virus Infection) OR (Hepatitis B virus) OR (B virus, Hepatitis) OR (Hepatitis B viruses) OR (Viruses, Hepatitis B) OR (Hepatitis Virus, Homologous Serum) OR (Hepatitis B Surface Antigens) OR (Hepatitis B Surface Antigen) OR (HBsAg) OR (Hepatitis B, Chronic) OR (Chronic Hepatitis B Virus Infection) OR (Chronic Hepatitis B) OR (Hepatitis B Virus Infection, Chronic)) AND ((Semen Analysis) OR (Semen Analyses) OR (Semen Quality Analysis) OR (Analyses, Semen Quality) OR (Quality Analyses, Semen) OR (Semen Quality Analyses) OR (Semen Quality) OR (Qualities, Semen) OR (Quality, Semen) OR (Semen Qualities) OR (Analysis, Semen Quality))) AND (1900/01/01:2023/08/01[dp])  Present the full search strategies for all databases, registers and websites, including any filters and limits used. |  |
| Selection process | 8 | YTX and KG authors independently performed retrieval, screening independently. Any disagreements were solved by consultation or the decision of another author (HWW or HM). Studies that complied with the inclusion criteria were separately read and screened in full.  Specify the methods used to decide whether a study met the inclusion criteria of the review, including how many reviewers screened each record and each report retrieved, whether they worked independently, and if applicable, details of automation tools used in the process. |  |
| Data collection process | 9 | YTX and KG authors independently performed data extraction tasks independently. Any disagreements were solved by consultation or the decision of another author (HWW or HM).  Specify the methods used to collect data from reports, including how many reviewers collected data from each report, whether they worked independently, any processes for obtaining or confirming data from study investigators, and if applicable, details of automation tools used in the process. |  |
| Data items | 10a | semen quality parameters (including semen volume, sperm concentration, total sperm count, sperm morphology, sperm motility, and sperm progressive motility)  List and define all outcomes for which data were sought. Specify whether all results that were compatible with each outcome domain in each study were sought (e.g. for all measures, time points, analyses), and if not, the methods used to decide which results to collect. |  |
|  | 10b | Included characteristics of study (first author, publication date, country, type of study) and characteristics of participant (recruitment period, number, age, use of assisted reproductive technology )  List and define all other variables for which data were sought (e.g. participant and intervention characteristics, funding sources). Describe any assumptions made about any missing or unclear information. |  |
| Study risk of bias assessment | 11 | We used RevMan software (version 5.3) to create a risk of bias (quality) assessment chart. Because the articles we included were observational (case-control and cohort studies) rather than randomized controlled trials, we used the Newcastle–Ottawa Scale (NOS) to assess the risk of bias [11, 12]. The NOS evaluates study quality in the following three categories: (1) study population selection (0 to 4 points); (2) comparability between groups (0 to 2 points); (3) outcome measurements (0 to 3 points). The NOS scores range from 0 to 9 (low quality, ≤ 5 points; medium quality, 6 to 7 points; high quality, 8 to 9 points). Two authors (YTX and KG) independently scored studies using the NOS, and any controversy were solved by consensus or another author (HWW). We did not exclude studies from our analysis due to low quality evaluation scores.  Specify the methods used to assess risk of bias in the included studies, including details of the tool(s) used, how many reviewers assessed each study and whether they worked independently, and if applicable, details of automation tools used in the process. |  |
| Effect measures | 12 | Compared with the semen quality of HBV-negative men, HBV infection had a negative association with semen volume (MD: −0.20 mL, 95%CI: −0.32 to −0.09, P = 0.0004), sperm concentration (MD: −4.46×106/mL, 95%CI: −7.09 to −1.84, P = 0.0009), sperm morphology (MD: −2.49%, 95%CI: −4.35 to −0.64, P = 0.008), sperm motility (MD: −6.85%, 95%CI: −11.53 to −2.18, P = 0.004), and sperm progressive motility (MD: −6.63%, 95%CI: −10.24 to −3.02, P = 0.0003). However, HBV infection had no significant association with total sperm count (MD: −31.50×106, 95%CI: −74.11 to 11.10, P = 0.15).  Specify for each outcome the effect measure(s) (e.g. risk ratio, mean difference) used in the synthesis or presentation of results. |  |
| Synthesis methods | 13a | Tabulating the study intervention characteristics as table 1.  Describe the processes used to decide which studies were eligible for each synthesis (e.g. tabulating the study intervention characteristics and comparing against the planned groups for each synthesis (item #5)). |  |
|  | 13b | We need to collect : mean, standard deviation and sample size of the experimental group; Mean, standard deviation and sample size of the control group.  Describe any methods required to prepare the data for presentation or synthesis, such as handling of missing summary statistics, or data conversions. |  |
|  | 13c | We used RevMan software (version 5.5) and Stata software (version 16.0) for this meta-analysis. Because the outcome observation index of our study was continuous variable data, the units were the same among all included studies, and the mean difference (MD) and its 95% confidence interval (CI) were used to express the effect size.  Describe any methods used to tabulate or visually display results of individual studies and syntheses. |  |
|  | 13d | We used RevMan software (version 5.5) and Stata software (version 16.0) for this meta-analysis. Because the outcome observation index of our study was continuous variable data, the units were the same among all included studies, and the mean difference (MD) and its 95% confidence interval (CI) were used to express the effect size.  Describe any methods used to synthesize results and provide a rationale for the choice(s). If meta-analysis was performed, describe the model(s), method(s) to identify the presence and extent of statistical heterogeneity, and software package(s) used. |  |
|  | 13e | We conducted a subgroup analysis comparing participants in China and other countries  Describe any methods used to explore possible causes of heterogeneity among study results (e.g. subgroup analysis, meta-regression). |  |
|  | 13f | We did a sensitivity analysis, eliminating one study at a time to investigate the sources of heterogeneity  Describe any sensitivity analyses conducted to assess robustness of the synthesized results. |  |
| Reporting bias assessment | 14 | Egger’s test was used to detect publication bias of included studies, a P-value < 0.1 indicated significant publication bias. |  |
| Certainty assessment | 15 | We used RevMan software (version 5.3) to create a risk of bias (quality) assessment chart. Because the articles we included were observational (case-control and cohort studies) rather than randomized controlled trials, we used the Newcastle–Ottawa Scale (NOS) to assess the risk of bias  Describe any methods used to assess certainty (or confidence) in the body of evidence for an outcome. |  |
| **RESULTS** | | |  |
| Study selection | 16a | 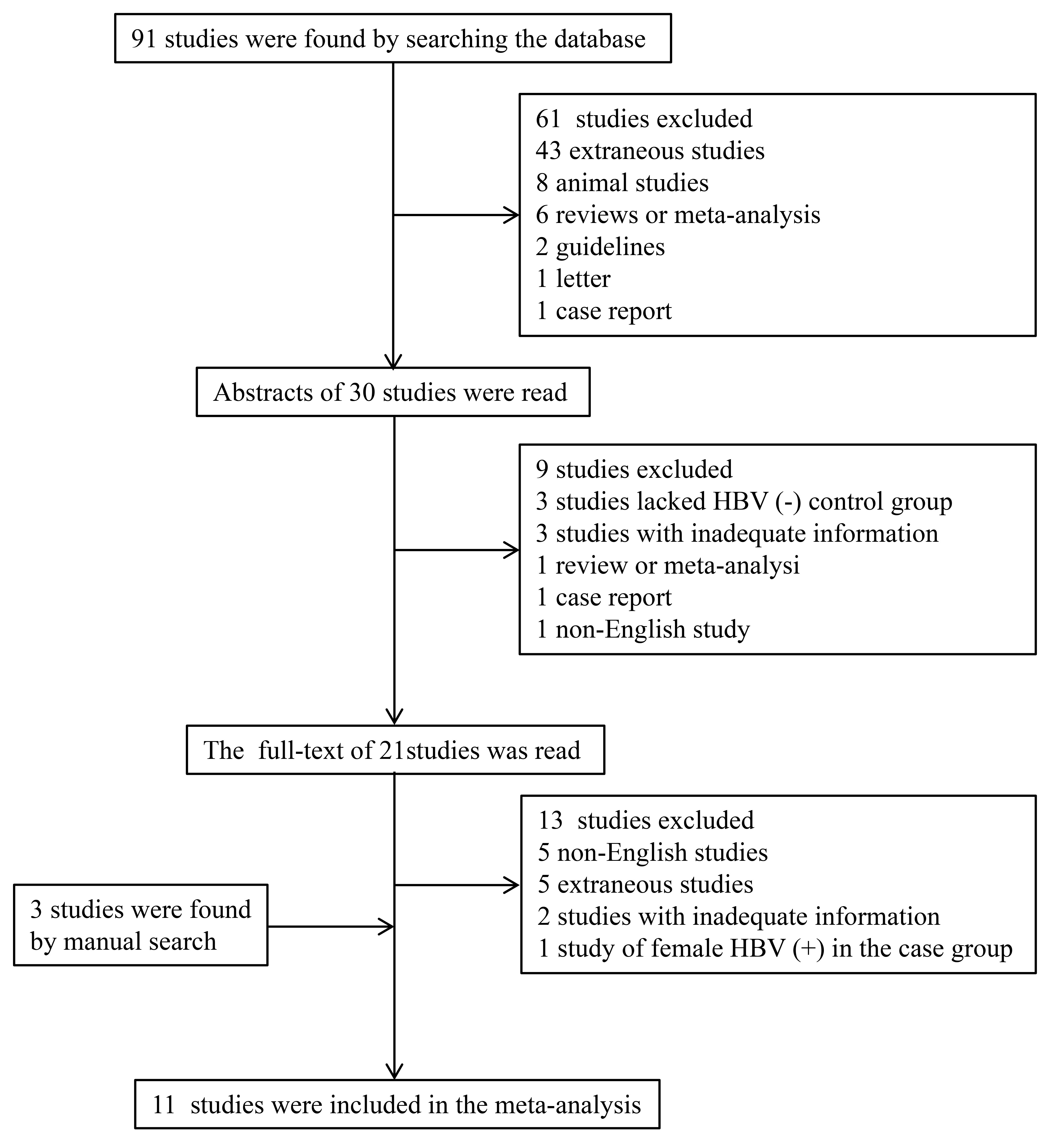 |  |
|  | 16b | The participants also suffered from HCV, Treponema pallidum (TP), human immunodeficiency virus, or herpes simplex virus  Cite studies that might appear to meet the inclusion criteria, but which were excluded, and explain why they were excluded. |  |
| Study characteristics | 17 | \| **Study** \| **Year published** \| **Country** \| **Study design** \| **Use of ART** \| **Recruitment Period** \| **HBV(+) group** \| \| **Control group** \| \| \| --- \| --- \| --- \| --- \| --- \| --- \| --- \| --- \| --- \| --- \| \| **Patients** \| **Age (years)** \| **Patients** \| **Age (years)** \| \| Bu et al \| 2014 \| China \| cohort study \| Yes \| 2010-2012 \| 20 \| 36.7±7.2 \| 257 \| 36±7.4 \| \| F. Lorusso et al \| 2009 \| Italy \| Case control \| Yes \| 2004-2008 \| 30 \| 36.9±6.1 \| 130 \| 36±4.1 \| \| G. Cito et al \| 2019 \| Italy \| cohort study \| Yes \| 2011-2018 \| 66 \| 36.7±6.5 \| 68 \| 38.7±4.4 \| \| Karamolahi et al \| 2019 \| Iran \| Case control \| Unclear \| 2003-2014 \| 112 \| 36.7±7.5 \| 112 \| 34.4±7.8 \| \| P. Oger et al \| 2011 \| France \| Case control \| Yes \| 2005-2008 \| 32 \| 34.7±5.0 \| 64 \| 35.3±6.3 \| \| Qian et al \| 2016 \| China \| Case control \| Unclear \| 2015-2015 \| 30 \| ... \| 30 \| ... \| \| Shi et al \| 2014 \| China \| Case control \| Yes \| 2008-2012 \| 136 \| 32.9±4.7 \| 272 \| 32.6±5.0 \| \| VCY Lee et al \| 2010 \| China \| Case control \| Yes \| 2004-2008 \| 154 \| ... \| 1473 \| ... \| \| Wang et al \| 2021 \| China \| Case control \| Yes \| 2016-2020 \| 227 \| 34.8±4.9 \| 454 \| 34.7±4.7 \| \| Yakass, M.B et al \| 2016 \| Ghana \| cohort study \| Yes \| 2013-2015 \| 27 \| ... \| 196 \| ... \| \| Zhou et al \| 2011 \| China \| Case control \| Yes \| 2008-2009 \| 457 \| 33.4±4.8 \| 459 \| 33.5±4.8 \|   Cite each included study and present its characteristics. |  |
| Risk of bias in studies | 18 | \| **Study** \| **Newcaste-Ottawa score** \| \| --- \| --- \| \| \| Bu et al \| Medium (6 points) \| \| F. Lorusso et al \| Low (5 points) \| \| G. Cito et al \| High (8 points) \| \| Karamolahi et al \| High (8 points) \| \| P. Oger et al \| Medium (7 points) \| \| Qian et al \| Medium (6 points) \| \| Shi et al \| Medium (7 points) \| \| VCY Lee et al \| Medium (6 points) \| \| Wang et al \| High (8 points) \| \| Yakass, M.B et al \| Medium (6 points) \| \| Zhou et al \| High (8 points) \| |  |
| Results of individual studies | 19 |    |  |
| Results of syntheses | 20a | Six studies involving 2,292 participants analyzed the association between HBV infection with semen volume.The heterogeneity test did not detect heterogeneity between studies (*P* = 0.98, *I*^2^ = 0%).  Nine studies involving 4,422 participants analyzed the association between HBV infection with sperm concentration. The heterogeneity test showed that the included studies were moderately heterogeneous (*P* = 0.07, *I*^2^ = 45%).  Three studies involving 1,274 participants analyzed the association between HBV infection with total sperm count. There was substantial heterogeneity in the included studies (*P* = 0.02, *I*^2^ = 73%).  Nine studies involving 3,730 participants analyzed the association between HBV infection with sperm morphology. The included studies were considerably heterogeneous (*P* < 0.00001, *I*^2^ = 97%).  Four studies involving 657 participants analyzed the association between HBV infection with sperm motility. The included studies had substantial heterogeneity (*P* = 0.03, *I*^2^ = 65%).  Ten studies involving 4,582 participants analyzed the association between HBV infection with sperm progressive motility. The included studies were considerably heterogeneous (*P* < 0.00001, *I*^2^ = 87%).  For each synthesis, briefly summarise the characteristics and risk of bias among contributing studies. |  |
|  | 20b | HBV infection had significant association with semen volume (MD: −0.20 mL, 95%CI: −0.32 to −0.09, *P* = 0.0004).  HBV infection had significant with sperm concentration (MD: −4.46×10^6^/mL, 95%CI: −7.09 to −1.84, *P* = 0.0009).  HBV infection had no significant association with total sperm count (MD: −31.50×10^6^, 95%CI: −74.11 to 11.10, *P* = 0.15).  HBV infection had significant with sperm morphology (MD: −2.49%, 95%CI: −4.35 to −0.64, *P* = 0.008).  HBV infection had significant with sperm motility (MD: −6.85%, 95%CI: −11.53 to −2.18, *P* = 0.004).  HBV infection had significant with sperm progressive motility (MD: −6.63%, 95%CI: −10.24 to −3.02, *P* = 0.0003).  Present results of all statistical syntheses conducted. If meta-analysis was done, present for each the summary estimate and its precision (e.g. confidence/credible interval) and measures of statistical heterogeneity. If comparing groups, describe the direction of the effect. |  |
|  | 20c | The primary contributors to heterogeneity were the confounding effects of clinical factors and variations in statistical methodologies. Heterogeneity in clinical factors may arise from variables such as age, gender, race, and disease severity. Regarding statistical methods, some studies employ standard deviation while others utilize standard error, potentially introducing heterogeneity in analytical approaches.  Present results of all investigations of possible causes of heterogeneity among study results. |  |
|  | 20d | Six studies analyzed the association between HBV infection with semen volume.The sensitivity analysis showed low sensitivity and relatively stable results.  Nine studies analyzed the association between HBV infection with sperm concentration. The sensitivity analysis showed low sensitivity and relatively stable results.  Three studies analyzed the association between HBV infection with total sperm count. The sensitivity was low, and the results were relatively stable.  Nine studies analyzed the association between HBV infection with sperm morphology. The sensitivity analysis showed low sensitivity and relatively stable results.  Four studies analyzed the association between HBV infection with sperm motility. The sensitivity analysis showed low sensitivity, and the result was relatively stable.  Ten studies analyzed the association between HBV infection with sperm progressive motility. The sensitivity analysis showed low sensitivity, and the result was relatively stable.  Present results of all sensitivity analyses conducted to assess the robustness of the synthesized results. |  |
| Reporting biases | 21 | Six studies analyzed the association between HBV infection with semen volume.The sensitivity analysis showed low sensitivity and relatively stable results. No publication bias was detected.  Nine studies analyzed the association between HBV infection with sperm concentration. No publication bias was detected.  Three studies analyzed the association between HBV infection with total sperm count. No publication bias was detected.  Nine studies analyzed the association between HBV infection with sperm morphology. No publication bias was detected.  Four studies analyzed the association between HBV infection with sperm motility. No publication bias was detected.  Ten studies analyzed the association between HBV infection with sperm progressive motility. Publication bias was detected.  Present assessments of risk of bias due to missing results (arising from reporting biases) for each synthesis assessed. |  |
| Certainty of evidence | 22 | HBV infection had a negative association with semen volume (MD: −0.20 mL, 95%CI: −0.32 to −0.09, *P* = 0.0004).  HBV infection had a negative association with sperm concentration (MD: −4.46×10^6^/mL, 95%CI: −7.09 to −1.84, *P* = 0.0009).  HBV infection had no significant association with total sperm count (MD: −31.50×10^6^, 95%CI: −74.11 to 11.10, *P* = 0.15).  HBV infection had a negative association with sperm morphology (MD: −2.49%, 95%CI: −4.35 to −0.64, *P* = 0.008).  HBV infection had a negative association with sperm motility (MD: −6.85%, 95%CI: −11.53 to −2.18, *P* = 0.004).  HBV infection had a negative association with sperm progressive motility (MD: −6.63%, 95%CI: −10.24 to −3.02, *P* = 0.0003).  Present assessments of certainty (or confidence) in the body of evidence for each outcome assessed. |  |
| **DISCUSSION** | | |  |
| Discussion | 23a | The results showed that HBV infection had negative association with sperm concentration, motility, morphology, and semen volume but no significant association with total sperm count.  Provide a general interpretation of the results in the context of other evidence. |  |
|  | 23b | First of all, the number of studies included was not large enough. Although only 11 studies were included, semen parameters were reflected in many aspects, including count, motility, and morphology, and not all studies reported all semen parameters. Second, there was no control for confounding factors. In particular, no subgroup classification of infertile and fertile individuals. Other factors that may influence semen parameters, including race, age, body mass index, medical history, dietary habits, and lifestyle, were not controlled for in some studies. Third, all included studies were retrospective cohort or case–control studies, making it impossible to prove a causal relationship.  Discuss any limitations of the evidence included in the review. |  |
|  | 23c | Some articles were of poor quality and lacked detailed inclusion and exclusion criteria.We did not exclude studies from our analysis due to low quality evaluation scores.  Discuss any limitations of the review processes used. |  |
|  | 23d | HBV infection had a negative association with sperm concentration, motility, morphology, and semen volume. However, The association between HBV and total sperm count remain unclear. This meta‑analysis suggests that we should pay attention to the adverse effect of HBV on sperm quality, and several studies have reported the relevant mechanisms. Further, clarifying the relationship between HBV infection and male infertility is necessary to provide theoretical guidance for the treatment of male HBV infection with infertility.  Discuss implications of the results for practice, policy, and future research. |  |
| OTHER INFORMATION | | |  |
| Registration and protocol | 24a | This [research](javascript:;) was conducted according to the PROSPERO guideline (Registration ID CRD42022311270) by Yuting Xu.  Provide registration information for the review, including register name and registration number, or state that the review was not registered. |  |
|  | 24b | https://www.crd.york.ac.uk/prospero/  Indicate where the review protocol can be accessed, or state that a protocol was not prepared. |  |
|  | 24c | Not applicable  Describe and explain any amendments to information provided at registration or in the protocol. |  |
| Support | 25 | National Natural Science Foundation of China (Grant No.82160281)  Describe sources of financial or non-financial support for the review, and the role of the funders or sponsors in the review. |  |
| Competing interests | 26 | Not applicable  Declare any competing interests of review authors. |  |
| Availability of data, code and other materials | 27 | All the data openly available in a public repository, and can be found in the database.  Report which of the following are publicly available and where they can be found: template data collection forms; data extracted from included studies; data used for all analyses; analytic code; any other materials used in the review. |  |

*From:*  Page MJ, McKenzie JE, Bossuyt PM, Boutron I, Hoffmann TC, Mulrow CD, et al. The PRISMA 2020 statement: an updated guideline for reporting systematic reviews. BMJ 2021;372:n71. doi: 10.1136/bmj.n71

For more information, visit: <http://www.prisma-statement.org/>
